# The Role of Flagella in *Clostridium difficile* Pathogenesis: Comparison between a Non-Epidemic and an Epidemic Strain

**DOI:** 10.1371/journal.pone.0073026

**Published:** 2013-09-23

**Authors:** Soza T. Baban, Sarah A. Kuehne, Amira Barketi-Klai, Stephen T. Cartman, Michelle L. Kelly, Kim R. Hardie, Imad Kansau, Anne Collignon, Nigel P. Minton

**Affiliations:** 1 Clostridia Research Group, NIHR Biomedical Research Unit in GI Disease, Centre for Biomolecular Sciences, University of Nottingham, Nottingham, United Kingdom; 2 Faculté de Pharmacie, EA4043, Université Paris-Sud, Châtenay-Malabry, France; Institute Pasteur, France

## Abstract

*Clostridium difficile* is a major cause of healthcare-associated infection and inflicts a considerable financial burden on healthcare systems worldwide. Disease symptoms range from self-limiting diarrhoea to fatal pseudomembranous colitis. Whilst *C. difficile* has two major virulence factors, toxin A and B, it is generally accepted that other virulence components of the bacterium contribute to disease. *C. difficile* colonises the gut of humans and animals and hence the processes of adherence and colonisation are essential for disease onset. Previously it has been suggested that flagella might be implicated in colonisation. Here we tested this hypothesis by comparing flagellated parental strains to strains in which flagella genes were inactivated using ClosTron technology. Our focus was on a UK-outbreak, PCR-ribotype 027 (B1/NAP1) strain, R20291. We compared the flagellated wild-type to a mutant with a paralyzed flagellum and also to mutants (*fliC*, *fliD* and *flgE*) that no longer produce flagella *in vitro* and *in vivo*. Our results with R20291 provide the first strong evidence that by disabling the motor of the flagellum, the structural components of the flagellum rather than active motility, is needed for adherence and colonisation of the intestinal epithelium during infection. Comparison to published data on 630Δ*erm* and our own data on that strain revealed major differences between the strains: the R20291 flagellar mutants adhered less than the parental strain *in vitro*, whereas we saw the opposite in 630Δ*erm*. We also showed that flagella and motility are not needed for successful colonisation *in vivo* using strain 630Δ*erm*. Finally we demonstrated that in strain R20291, flagella do play a role in colonisation and adherence and that there are striking differences between *C. difficile* strains. The latter emphasises the overriding need to characterize more than just one strain before drawing general conclusions concerning specific mechanisms of pathogenesis.

## Introduction

*Clostridium difficile* is the principle cause of hospital acquired antibiotic associated diarrhoea in North America and Europe. The morbidity and mortality rates of nosocomial *Clostridium difficile* infection (CDI) continue to rise, particularly following the global emergence of epidemic *C. difficile* strains (027/BI/NAP1) [1], [2]. The two main virulence factors of CDI are the large clostridial toxins A and B [3]–[6]. However, other factors undoubtedly contribute to disease. Gut colonisation is a prerequisite for CDI, yet little is known of the mechanisms involved. The mucosal surface carpeting the intestinal epithelium is the main site of host-pathogen interaction, in which this organism must both evade the immune response and interact with enterocytes and adhere to specific surface molecules. *C. difficile* possesses multiple putative surface adhesins, potentially functioning as colonisation factors, including cell surface-associated proteins (S-layer and SLPs), fibronectin-binding protein FbpA, proteases such as Cwp84, hydrolytic enzymes, heat-shock proteins such as GroEl [7]–[9] and flagellar cap FliD and flagellin FliC structural components [10]. FliD and FliC are both components of the bacterial flagellum, an important multi-purpose structure that has diverse biological functions to favour bacterial survival and host colonisation [11].

For most gastrointestinal pathogens, flagella and flagellum-mediated motility are recognised as essential virulence factors, rendering the pathogen more capable of moving towards the site of colonisation. For instance, the intestinal enteric pathogens *Listeria monocytogenes* [12] and *Vibrio anguillarum* [13] strictly require functional flagellum-mediated motility to invade their hosts and establish successful colonisation. Flagellum-mediated motility is also an essential virulence factor required by *Helicobacter pylori* to colonise the stomach [14]. Pathogen survival *in vivo* can be enhanced through the formation of complex communities known as biofilms, and flagella have been shown to play a role in the formation and development of biofilms in a number of pathogens [15], [16], most recently *C. difficile* [17].

A role for flagellum components as adhesins mediating bacterial attachment to host cell surfaces noted above is not restricted to *C. difficile*. For example, the flagellum of enteropathogenic *Escherichia coli* contributes to adherence to epithelial cells independent of flagellum-mediated motility [18]. Moreover, Flagellin (FliC) and the flagellar cap protein (FliD) of *Pseudomonas aeruginosa*, are associated with adherence and colonisation of the respiratory tract through recognition of mucin (Muc1), an abundant component protein of airway mucus [19]. In the past, the absence of effective genetic tools led to the deployment of restricted and indirect techniques in attempting to define the potential role of flagella in adherence and colonisation of *C. difficile*. Tasteyre *et al.* [20] observed that the presence of flagella increases the capacity of *C. difficile* to associate with the intestinal epithelial tissue. The flagellated, motile *C. difficile* attach more efficiently to the caecal wall of axenic mice than non-flagellated strains of the same serogroup. Moreover, in a separate analysis purified recombinant flagellar cap (FliD) and flagellin (FliC) proteins were shown to attach to tissue culture cells [10]. These studies led to the conclusion that flagellin and the flagellar cap may serve as one of the multiple cell-surface adhesins of *C. difficile*. This contradicted an earlier study [21] of the flagella of clinical *C. difficile* strains which concluded flagella played no role in adherence, since the antiserum that was raised against the purified recombinant flagellin did not inhibit adherence to cultured cells.

Recently a paper was published by Dingle *et al.* [22] investigating the importance of flagella in the virulence of *C. difficile* strain 630Δ*erm*. For the first time they were able to construct isogenic mutants in *fliC* and *fliD* using ClosTron technology [23]. Interestingly they found that the flagella mutants adhered more strongly to Caco2 cells *in vitro* and showed increased toxicity *in vitro* and *in vivo* tested in the hamster model of infection. Whilst the majority of *C. difficile* isolates appear to produce flagella, a high degree of variation of flagella-related gene content is evident [10], [24]. It is therefore of value to extend these studies to further strains before drawing any general conclusions as to the involvement of flagella and motility in the virulence of this bacterium. In particular, investigation of the role of flagella in more relevant epidemic strains is required. In the present study we have focussed on the epidemic 027/BI/NAP1 strain, R20291. Our aim was to elucidate the mechanism by which flagella contribute to *C. difficile* adhesion to human intestinal epithelial cells and the potential role of motility in colonisation of the intestinal tract in mice through the comparative analysis of directed mutants in specific flagellar genes in both *C. difficile* 630Δ*erm* and R20291.

## Results and Discussion

### Construction and phenotypic characterizations of flagellar-associated mutants of *C. difficile* 630Δ*erm* and R20291

**Figure 1:**
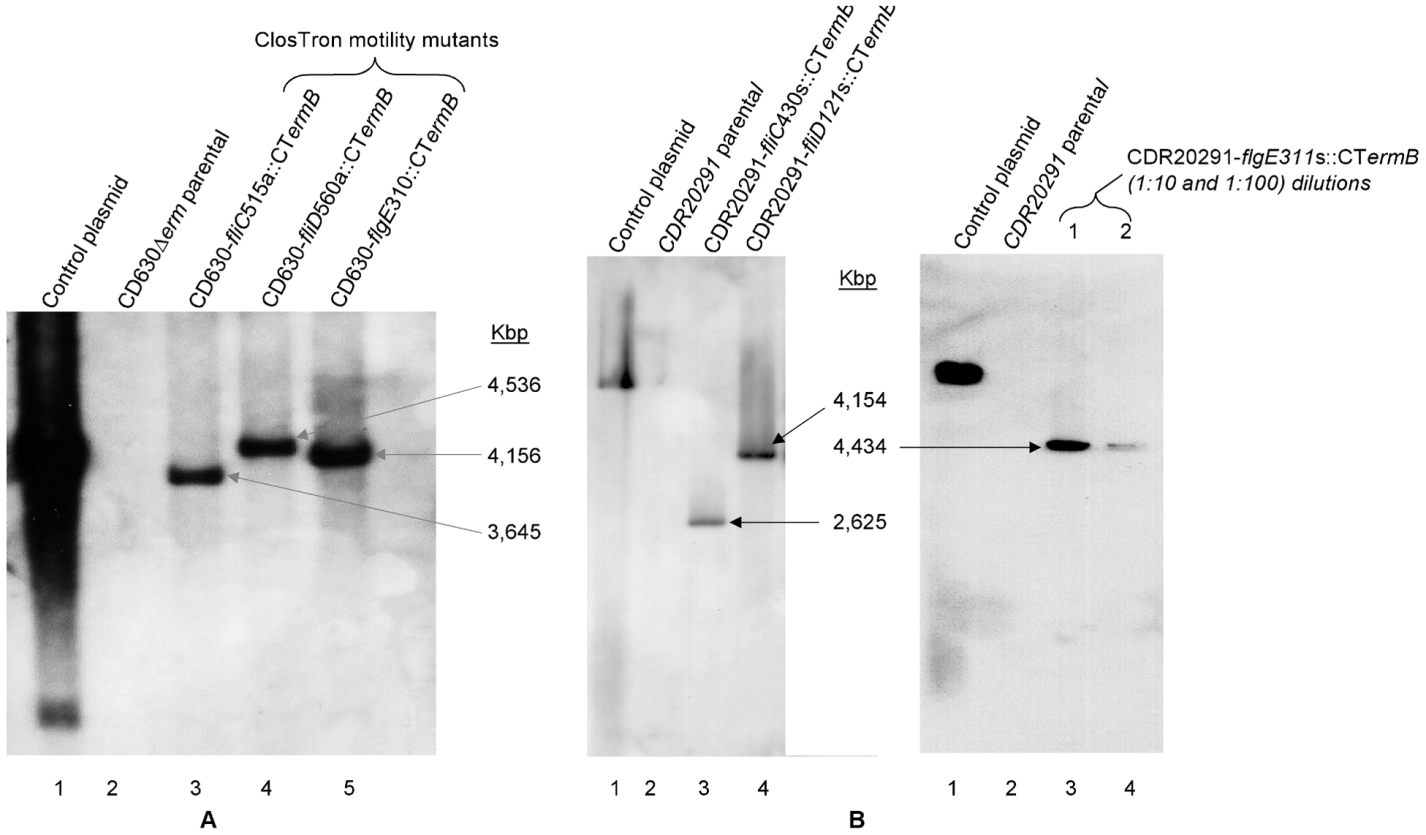
Confirmation of mutant construction by Southern blotting analysis. Southern hybridization of the ClosTron flagellar mutants of *C. difficile* 630Δ*erm* (a) and R20291 (b) strains using the intron probe to *ermB* in order to demonstrate the presence of a single intron insertion in mutants. Chromosomal genomic DNA of all strains was digested overnight with *Hin*dIII restriction enzyme, along with the retargeting ClosTron plasmid DNA. The DIG-labelled probe was designed to target the ErmRAM sequence and as expected, a single copy of ErmRAM was detected in each mutant. The control plasmid represents the ClosTron retargeting *fliC* vector (pMTL007C-E2:*fliC*- 430s-R20291) (lane 1); wild-type represents the *C. difficile* 630Δ *erm* and R20291 strains (lane 2); lanes 3 to 5 represents three isogenic ClosTron motility mutants. The arrow shows the obtained single group II intron insertion band of the expected size in each mutant. (**b**) The *flgE* mutant or *C. difficile* R20291, in which the occurrence of only single intron insertion in the D630-*flgE*::CT*ermB* mutant was confirmed using 1∶10 (lane 3) and 1∶100 (lane 4) dilutions of digested chromosomal DNA of this mutant hybridized to intron probe. The arrow shows the obtained single group II intron insertion band of the expected size in the mutant.

**Figure 2:**
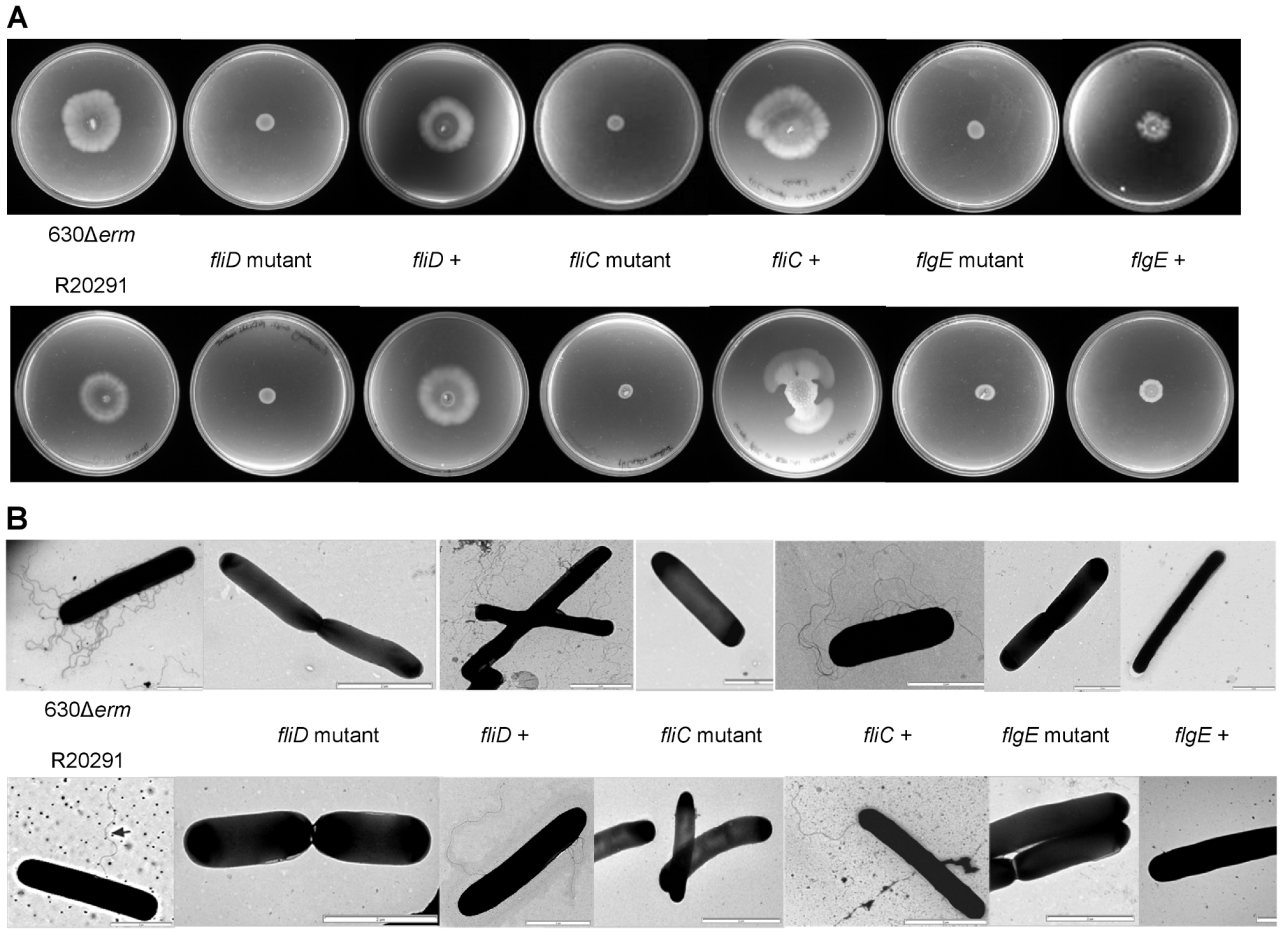
Phenotypic assays of parental strains and flagella mutants. (**A**) Swimming motility of the parental strains and flagella mutants and complemented mutants. (**B**) Transmission electron microscope (TEM) examination of wild-types and flagellar mutant. Cells were negatively strained with 0.4% URA and visualized by TEM (Scale bar represents 2 µm).

In order to analyse the role of flagella in the pathogenesis of *C. difficile* three different mutants were made in both R20291 and 630Δ*erm*. Using ClosTron technology [25] we insertionally inactivated *fliD*, coding for the flagella cap, *fliC*, encoding flagellin and *flgE*, encoding the hook protein. The resultant mutants CRG3357 (Cdi630Δ*erm*-*fliC*515a::CT), CDR3356 (Cdi630Δ*erm*-*fliD*560a::CT). CRG3355 (Cdi630Δ*erm*-*flgE*310s::CT), CRG3351 (CdiR20291-*fliC*430s::CT), CRG3350 (CdiR20291-*fliD*121s::CT) and CRG3349 (CdiR20291-*flgE*311s::CT) were verified by PCR using a gene specific and an intron specific primer (Table S1) to amplify across the junction (Fig. S1) PCR products were also sequenced. Southern blot analysis using an intron specific probe confirmed that only one insertion had occurred in each mutant (Fig. 1). A Western blot probing against FliC with a polyclonal antibody revealed that none of the mutants produced flagellin, while it was readily detected in the parental strains (data not shown). Motility of the mutants was tested on swimming and swarming plates and compared to the parental strains. Whereas both 630Δ*erm* and R20291 showed motility on plates (Fig. 2a, extreme left handside) all three mutants in the respective strains were completely immobilized (Fig. 2a). The respective complementation plasmids pMTLSB-1 (*fliC*), pMTLSB-2 (*fliD*) and pMTLSB-3 (*flgE*) were introduced into each mutant. In each plasmid, the cloned gene had been placed under the transcriptional control of the promoter of the *fliC* gene (Materials and Methods). Plasmid pMTLSB-1 was introduced into the *fliC* mutants CRG3357 and CRG3351 and successfully complemented the motility phenotype. Likewise pMTLSB-2 introduced into CRG3356 and CRG3350, the *fliD* mutants, partially complemented that phenotype back to wild type. However complementation of CRG3355 and CRG3349 with pMTLSB-3 was only partially successful. This was most likely due to the fact that the *fliC* promoter controls gene expression in class three flagella genes, whereas *flgE* is transcribed as a class two gene. Disrupting the highly organised flagella assembly by inappropriate transcription signals might be an explanation why no full complementation was achieved with these constructs (Fig. 2a). The parental strains and derived mutants and complemented mutants, were all examined under the transmission electron microscope (TEM). Interestingly we observed a great difference between 630Δ*erm* and R20291. Whereas 630Δ*erm* was peritrichously flagellated, R20291 was monotrichously flagellated with only a single flagellum present on its surface. This most likely explains why R20291 displayed less motility than 630Δ*erm* in the swimming and swarming assays. No flagella were observed for any of the flagella mutants (Fig. 2b).

### Construction of a paralyzed flagellum

To address the question as to whether flagella act as adhesins or function merely as a motility apparatus we generated mutants in the motor genes *motA* and *B* and also in the rotor gene *fliG*. After analysis of these mutants via TEM and motility plates we noticed that these mutants were not able to swim or swarm and the TEM revealed a complete lack of flagella for the *motB* and the *fliG*-mutant and a 50% reduction for the *motA*-mutant (data not shown). This finding is in line with previously reported data in *Listeria monocytogenes* [12] which demonstrated that a complete disruption of motor genes leads to loss of flagella. We can thus conclude that these genes do not only play a role in the motor but also act as a structural or assembly component. A multiple alignment showed that the aspartate residue at position 23 was highly conserved. This residue has successfully been changed in Listeria to obtain a paralyzed flagellum. Using homologous recombination [26] we changed aspartate 23 to alanine in *C. difficile* R20291 and examined the mutant under the TEM and in motility assays (Fig. S2). The microscopy revealed a single flagellum, like the wild-type, but no motility was observed. This strain, R20291 *motB*:D23A, was used alongside the structural flagella mutants in *in vitro* adherence assays.

### The flagellum of R20291 is used as an adhesin to enhance bacterial association to human intestinal epithelial cells *in vitro*

**Figure 3:**
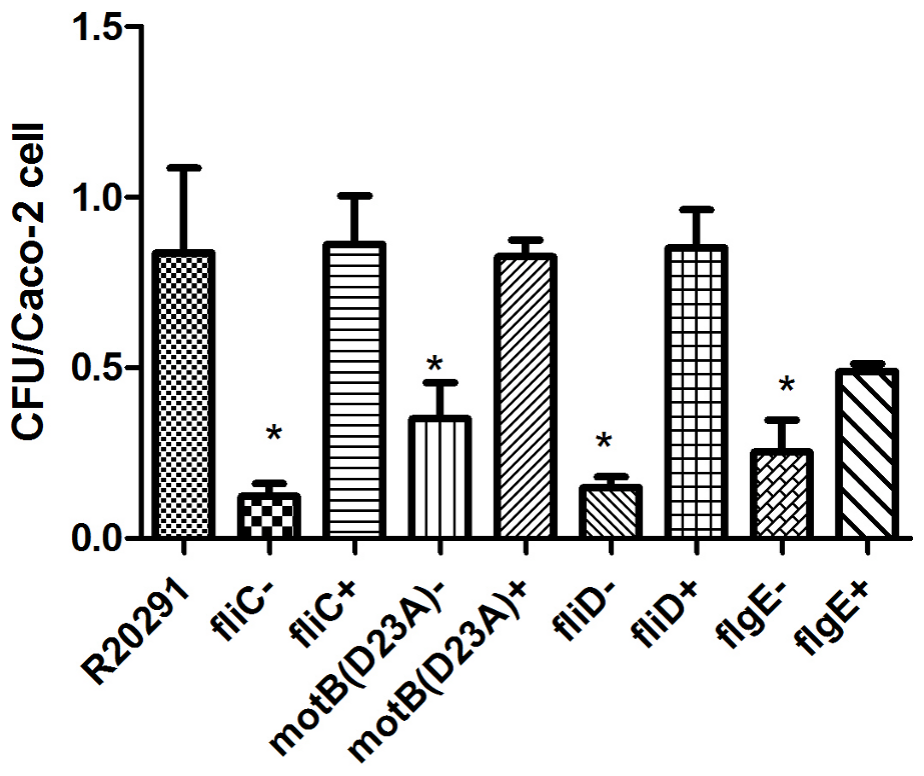
Adherence of *C. difficile* R20291 flagella mutants to human intestinal epithelial Caco-2 cell-line is impaired. Differentiated monolayers of Caco-2 cells were incubated with each of *C. difficile* R20291 wild-type strain, non-motile non-flagellated mutants (CRG430, CRG122, CRG2705), non-motile paralyzed-flagellated *motB* (D23A) mutant and the complemented strains (CRG-SB1, CRG-SB2, CRG-SB3, and CRG-SB4). Cell adherence level was measured by the bacterial adherence assay as described in Materials and Methods. The presented data are means ± standard errors of the means for three independent experiments. Statistically significant differences in flagellar mutants compared to *C. difficile* R20291 wild-type are represented by* for *P*<0.05.

The current literature describing the role of the *C. difficile* flagellum in adhesion is contradictory. In keeping with a previous report investing the adherence of the non-epidemic *C. difficile* strain 630Δ*erm* to Caco-2 cell [22], we found that both a *fliC* and a *fliD* mutant adhered more strongly than the wild-type. In contrast, the *flgE* mutant of 630Δ*erm* adhered less strongly than the wild-type, albeit the latter was not statistically significant (Fig. S3). We extended this investigation of adherence by examining the role of flagella in the epidemic strain R20291 for the first time. The adherence of the flagella mutants, paralyzed mutant, wild-type and complemented strains to Caco-2 cells was assayed (Fig. 3). All flagella mutants were less effective in adherence than the wild-type. In particular the *fliC*, *fliD* and *flgE*-mutant all adhered less than the paralyzed *motB*-mutant, which still adhered significantly less than the wild-type (p<0.05). These results indicate an important role for the flagella of R20291 in adherence and, moreover, indicate that the intact flagellar structure possesses adhesive properties that are independent of flagellum-mediated motility. These findings are in contrast to results obtained with strain 630Δ*erm* (this study and [22]), which adheres less effectively than the corresponding *fliC* and *fliD* flagella mutants. It is important to note that we did not observe any difference in growth kinetics between wild-type, mutants and complemented strains *in vitro* (data not shown).

### Assessment of importance of the flagellum of R20291 in intestinal colonisation of gnotobiotic mice

As flagella in R20291 seem to play a role in adherence we tested the wild-type, the *fliC*-mutant (CRG3351) and the paralyzed flagellum mutant (CRG1987) *in vivo* using the monoxenic mouse model for a single infection study and thereafter the dixenic mouse model in a competition experiment. We chose not to put the complemented strains through our animal models as plasmids are known to be unstable in *C. difficile* if antibiotic selection is not maintained. Results would hence be doubtful and as such these experiments were deemed unethical.

**Figure 4:**
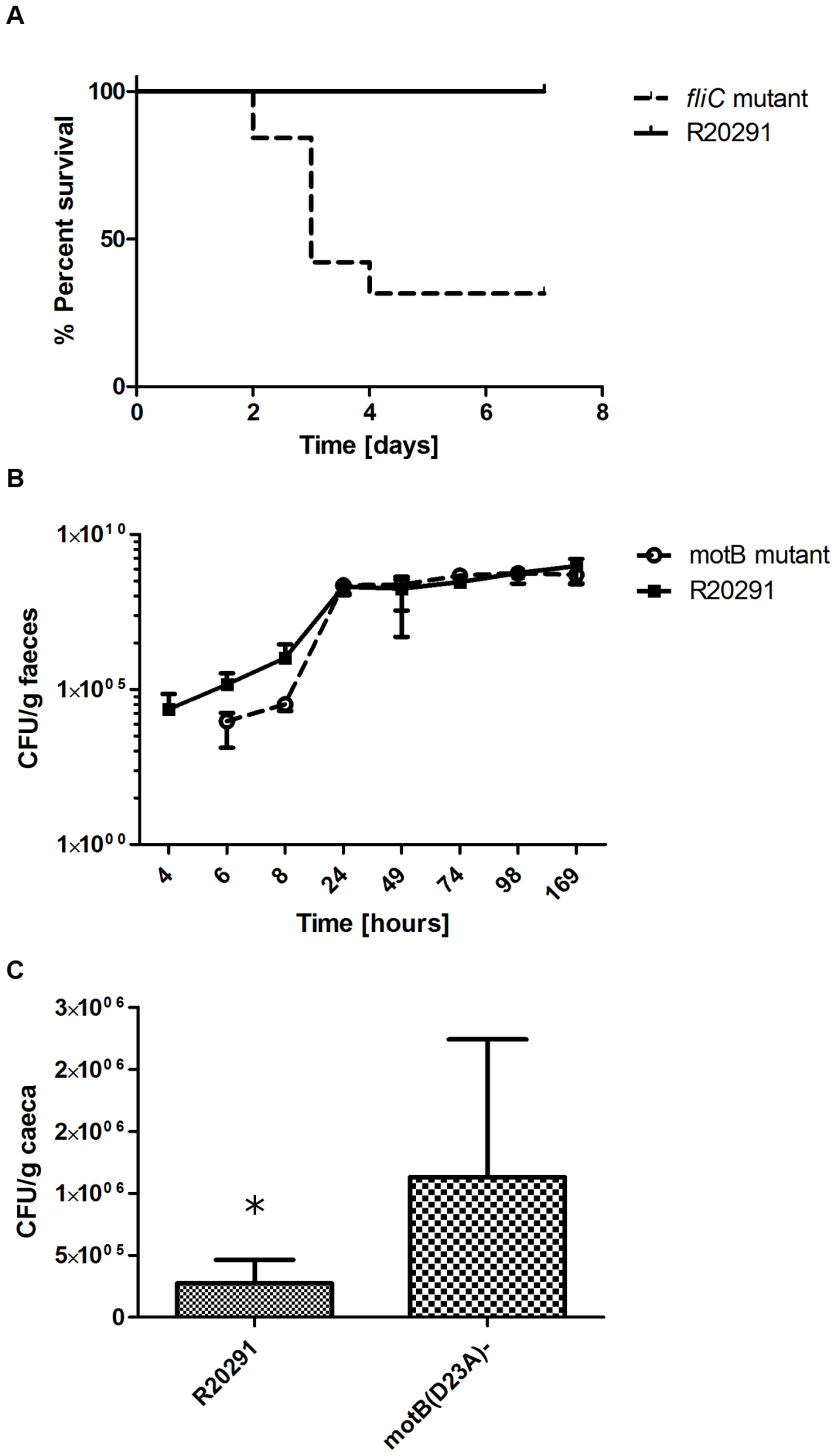
Role of *C. difficile* R20291 flagellum in intestinal colonisation of monoxenic mice. (**A**) Kaplan-Meier plot survival analysis of monoxenic mice infected with *C. difficile R20291* and *fliC* mutant, (**B**) Kinetic of intestinal implantation by the *C. difficile* R20291 wild-type strain and paralyzed-flagellated *motB* mutant. Mice (n = 6) were challenged with 1×10^7^ bacteria of single *C. difficile* strain. Faecal pellets were collected and processed at regular time intervals daily during a week to determine the kinetic of faecal shedding (CFU per g of faeces) for each of the two strains, as described in Materials and Methods. (**C**) Adherence of motile flagellated *C. difficile* R20291 wild-type strain and the paralyzed-flagellated *motB* mutant to mouse caecum. The entire caecum of each mouse was collected at day 7 post-infection, and processed for determination of adhered viable CFU to caecum tissue of mice as described in Materials and Methods. Results are presented as the number of adhered *C. difficile* per g of caeca. Statistically significant difference is indicated by *for *P*<0.05.

For our individual mouse infection study, we used a monoxenic mouse model. All 6 mice infected with the wild-type R20291 survived the entire experimental duration (7 days) without succumbing to *C. difficile* disease. Colonisation was monitored through faecal shedding kinetics and at the end of the experiment adherence to the caecum was measured. Surprisingly, 70% of mice infected with the *fliC*-mutant CRG3351 succumbed to *C. difficile* disease by four days (Fig. 4A). A comparison between the wild-type and the paralyzed mutant (*motB*(D23A)-) revealed no difference in faecal shedding (Fig. 4B) and significant difference in adherence to the caeca (Fig. 4C). These findings suggest that flagella-associated motility is not needed for successful colonisation and adherence in mice, although the structure of the flagellum itself may be important as an adhesin. Further investigation is necessary to understand the increased virulence of the *fliC*-mutant.

**Figure 5:**
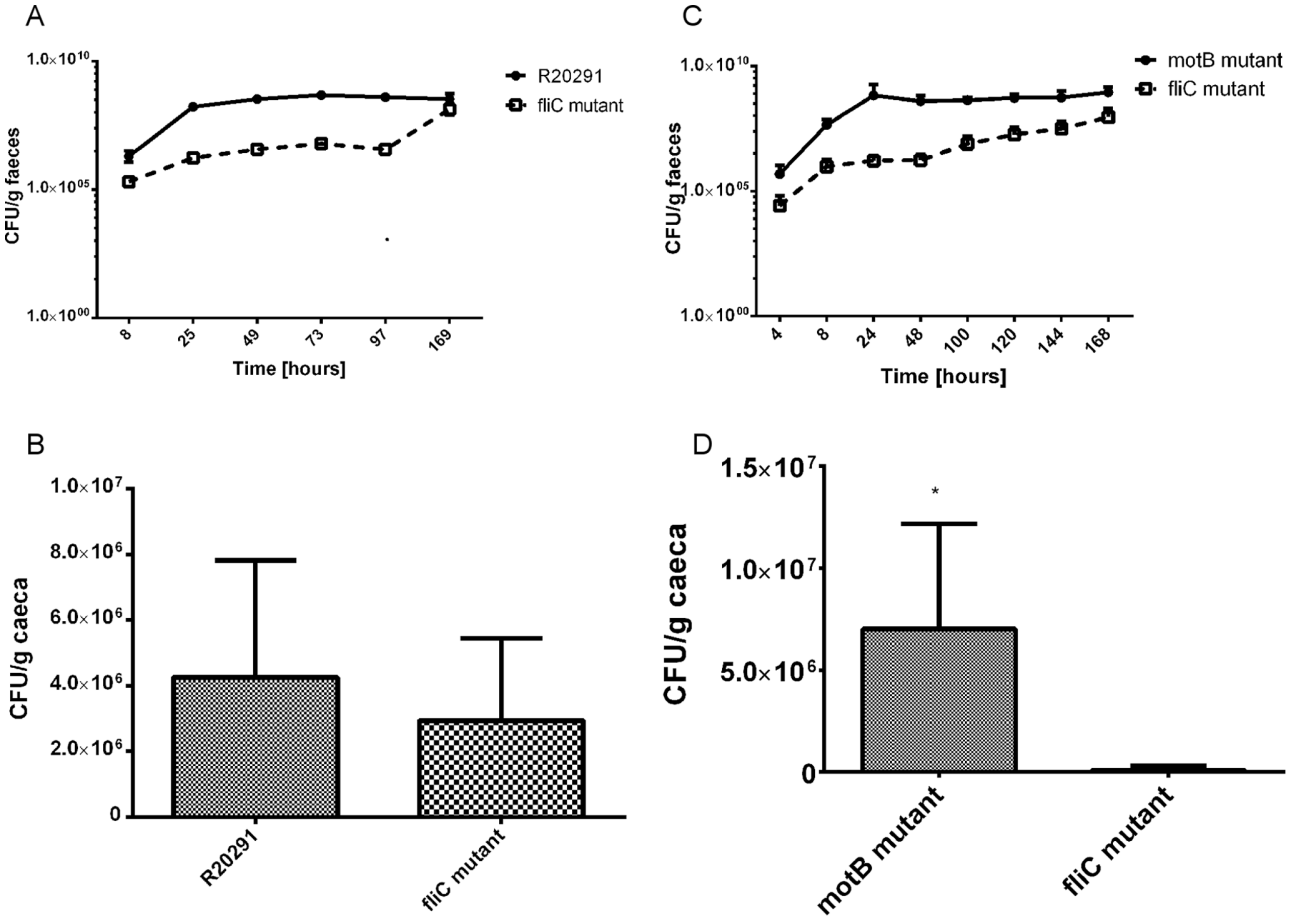
Role of *C. difficile* R20291 flagellum in intestinal colonisation of dixenic mice with non-motile non-flagellated *fliC* and paralyzed-flagellated *motB* mutants. (**A**) Kinetics of intestinal implantation by wild-type strain and non-motile non-flagellated *fliC* mutant. Each mouse was co-challenged with the 2 strains and the kinetics of faecal shedding was monitored by using the same protocol described for in the mono-axenic mice model, as outlined in Materials and Methods. (**B**) Caecal colonisation of motile flagellated *C. difficile* R20291 wild-type strain and the non-motile non-flagellated *fliC* mutant. (**C**) Kinetics of intestinal implantation by the paralyzed *motB* mutant and non-motile non-flagellated *fliC* mutant. Each mouse was co-challenged with the 2 strains and the kinetics of faecal shedding was monitored by using the same protocol described for in the mono-axenic mice model, as outlined in Materials and Methods. (**D**) Caecal colonisation of paralyzed flagellated *C. difficile* R20291 *motB* mutant and the non-motile non-flagellated *fliC* mutant. Statistically significant difference is shown by * for *P*<0.05.

To confirm the results obtained in single infection, and to further investigate the advantage for R20291 to possess flagella, we co-challenged mice with a 1∶1 inoculum of wild-type and *fliC*-mutant. The wild-type clearly out-competed the non-motile CRG3351 strain, showing increased colonisation resulting in higher levels of faecal shedding (Fig. 5A) and adherence to the caeca, albeit the latter was not significant (Fig. 5B). We then co-challenged mice with the *fliC*-mutant and the paralyzed mutant (1∶1). The paralyzed mutant out-competed the *fliC*-mutant in colonisation and adherence (Fig. 5C and 5D). These *in vivo* experiments confirm the role of flagella as an adhesin during colonisation and demonstrate that flagellum-mediated motility is neither important nor essential for virulence of *C. difficile* R20291.

Taken together these *in vitro* adherence and *in vivo* intestinal colonisation studies provide the first strong evidence that by disabling the motor of the flagellum, the structural components of the flagellum of *C. difficile* R20291 rather than active motility, is needed for adherence and colonisation of the intestinal epithelium during infection.

### Comparison between the epidemic strain R20291 and non-epidemic isolate 630Δ*erm*

Previous flagella studies in *C. difficile* focused on the non-epidemic strain 630Δ*erm*. As epidemic strains, particularly those from PCR-Ribotypes 027 and 078 are becoming increasingly more common, it is important to ascertain whether they behave the same as 630Δ*erm*. In this study we observed differences between the two parental strains used, which is consistent with the fact that differences in the flagella regions of R20291 and 630 have been described previously [24]. In contrast to the peritrichiously flagellated 630Δ*erm*, R20291 only displays a single flagellum and is comparatively less motile in motility assays. This can clearly be seen in figure 2A and B. When tested in adherence assays *in vitro*, 630 and 630Δ*erm* adhered significantly less to Caco-2 cells than R20291. Interestingly flagella mutants of R20291 showed decreased adherence whereas the same mutants in a 630 background adhered more than the wild-type (Fig. 3 and S3).

### *In vivo* colonisation of *C. difficile* 630Δ*erm* and the *fliC*-mutant CRG3357

Reminiscent of our murine infections with strain R20291 and the derived *fliC* mutant, it has been reported that hamsters succumb to disease quicker when infected with a *fliC* or a *fliD*-mutant of 630Δ*erm* [22]. Although, Dingle *et al.* [22] measured higher toxicity levels with the mutants than with the parental strain, they did not analyse colonisation over time within the hamster. We compared the parental strain 630Δ*erm* to the *fliC*-mutant, CRG3357, in three different mouse models, which are traditionally used to investigate colonisation over time and adherence. Firstly we infected groups of six human-microbiota-associated (HMA) mice with the respective strains. This model is closest to humans as the implanted microbiota is disturbed by antibiotics prior to the bacterial challenge. We did not find any difference between the colonisation kinetics or adherence to the caecum between the two groups (Fig. S4). The microbiota might mask some subtle differences between wild-type and mutant. We subsequently tested the strains in the monoxenic mouse model in a single infection study. Again the *fliC*-mutant colonised and adhered at the same level as the wild-type (Fig. S4) leading to the conclusion that flagella are not required for strain 630Δ*erm* to colonise mice. In order to detect any subtle differences between the strains we undertook a co-infection study, giving the mice a 1∶1 inoculum of wild-type and *fliC*-mutant. Although the kinetics remained generally equivalent, there were minor differences at several time points (Fig. S4), indicating a fitness advantage of the wild-type. Furthermore the *fliC*-mutant adhered significantly less to the mouse caecum than the wild-type (Fig. S4). In conclusion, these results show that flagella and motility are not required for *C. difficile* 630 to colonise mice; however, flagellum motility may contribute to the fitness of the bacterium and allow the wild-type strain to outcompete the *fliC*-mutant. Interestingly, other bacteria exhibit a similar contribution of flagella to their infection pathway. For example, competition of motility mutants with wild-type *H. pylori* during mixed infections results in greater attenuation than observed in independent challenges in mice. Moreover, a *fliC* mutant of UPEC has previously been shown [27], [28] to be at a competitive disadvantage during mixed infection with the parental strain in a dixenic mouse model, whilst the mutant and the wild-type colonised to levels that were not significantly different during an independent challenge.

### Toxin production of 630Δ*erm* and flagella mutants in comparison to R20291 and flagella mutants

**Figure 6:**
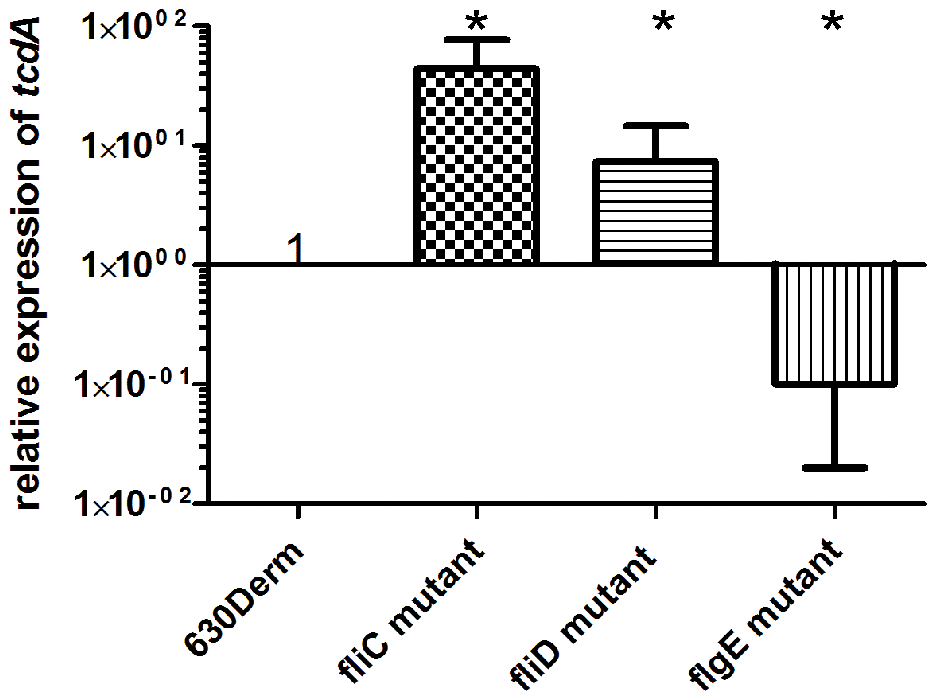
Quantitative Real Time PCR (qRT-PCR) analysis to assess *tcdA* expression in the flagellar structural mutants (*fliD*, *fliC* and *flgE*) compared to *C. difficile* 630Δ*erm* wild-type strain. The TcdA *(tcdA)* mRNA expression at late exponential-early stationary growth phase, 16 h growth time point. The *rpoA* mRNA was used as a reference. Each bar represents the average of two independent cultures. Error bars indicate the standard deviations. Asterisk (*) refers to significantly different from the wild-type (*P*<0.05).

Since increased toxicity was noted in the *fliC*- and *fliD*-630Δ*erm* flagella mutants [22], we measured the toxicity of a *flgE*-mutant in 630Δ*erm* (Fig. S5). In contrast to the *fliC* and *fliD*-mutant, the *flgE*-mutant showed reduced toxicity compared to the wild-type; this was however not statistically significant. Interestingly no difference in cytotoxicity was observed when comparing R20291 and its flagella mutants (data not shown). We performed glutamate dehydrogenase (GDH) assays in order to investigate whether increased cell lysis is responsible for more toxin being released in the *fliC* and *fliD*-mutants of 630Δ*erm*. GDH activity did not show any significant difference in any of the strains (data not shown) leading to the conclusion that cell lysis cannot explain the altered levels of toxins in the culture supernatant of the flagella mutants. In order to investigate whether the increased amount of toxicity in the mutants was due to altered levels of expressed toxin genes we performed qRT-PCR comparing the relative expression of *tcdA* (encoding for TcdA) in each flagellar mutant (*fliD*, *fliC* and *flgE*) to the parental strain 630Δ*erm*. Total cellular RNA was extracted during late exponential to early stationary phase of growth, after 16 h. The results of qRT-PCR analysis showed that the expression of the *tcdA* gene was strongly up-regulated in the *fliC* and *fliD*-mutants. The expression of *tcdA* was on average 44.1-fold greater in the *fliC* mutant and 7.4-fold greater in the *fliD*-mutant than in the wild-type strain (Fig. 6). These results confirmed that the high levels of toxin in the *fliD* and *fliC*-mutants were indeed due to increased expression of the *tcdA* gene. In the case of the *flgE*-mutant *tcdA* expression was on average 10-fold lower compared to the wild-type strain (Fig. 6).

Taken together, these observations provide experimental evidence that inactivation of flagellar cap (*fliD*), flagellin (*fliC*) and hook (*flgE*) genes have had an influence on the expression of *tcdA*. However, the mechanism underlying this regulatory relationship between the synthesis of flagella structure and regulation of toxin production is as yet unknown. The bacterial flagella system is tightly regulated in an ordered cascade, which uses a series of intermediate assembly stages as checkpoints. This tight regulatory control is required because the production of flagella and rotation are a metabolically costly undertaking for the cell, in which as many as 20,000 to 30,000 flagellin subunits are produced and their secretion outside of the cell and subsequent polymerization requires about 2% of the cell's energy expenditure. In flagella mutants, this metabolic energy is saved and can perhaps be used for other cell functions, such as production of TcdA and TcdB. On the other hand, we observed a decrease of toxin expression level in the *flgE*-mutant, which suggests a regulatory mechanism between flagella and toxin expression at least in 630Δ*erm*. Moreover, the GDH enzyme assay confirmed that the increase of toxin level in supernatants of the *fliD* and *fliC*-mutants was not due to an increase in cell lysis.

Recently a paper was published investigating the modulation of toxin transcription by the flagellar regulon [29]. The authors compared a *fliC*-mutant in 630Δ*erm* to the parental strain and a set of early flagella genes including *fliF*, *fliG* and *fliM*. As before they found that a *fliC*-mutant produces more toxin than the wild-type and in line with our findings the authors observed upregulation of *tcdA* expression. All the mutants in the early flagella genes showed a down-regulation of toxicity and *tcdA* expression comparable to our finding with the *flgE*-mutant. Furthermore they showed that these mutants exhibit less virulence in the hamster infection model. In addition to the transcription of *tcdA* they also examined *tcdB*, encoding TcdB, by qRT-PCR. Changes were more subtle, but might yet be important given the toxic effect of TcdB.

## Conclusions

This study compares the effect of flagella and flagellum-mediated motility between the non-epidemic *C. difficile* strain 630Δ*erm* and the UK outbreak strain R20291. R20291 produces only a single flagellum whereas 630Δ*erm* is peritrichously flagellated. It has been shown previously that flagella mutants in 630Δ*erm* [22] adhere more strongly to Caco-2 cells than the parental strain. In contrast, here we show that flagella mutants of R20291 adhere less strongly *in vitro* than the parental strain. Mice experiments using 630Δ*erm* wild-type and flagellar mutants revealed that flagella are not required for adherence and colonisation in this strain, but flagella-mediated motility might contribute to an overall fitness of the bacteria. An increase in toxicity was noted in strains in which the late flagellar genes (*fliC* and *fliD*) of 630Δ*erm* were mutated, a phenotype which was not apparently due to increased cell lysis. We confirmed by qRT-PCR that expression of the *tcdA* gene was up-regulated in these mutants. The *flgE*-mutant of 630Δ*erm* showed lower cytotoxicity and also a decreased expression of *tcdA*. These results were recently confirmed in an independent study [29] which analysed a *fliC*-mutant, as well as mutants in early flagellar genes. In the case of R20291, however, we did not see any change in toxicity between the wild-type and the flagellar mutants. Mice experiments showed that flagellum-mediated motility is not necessary for colonisation and adherence; however a paralyzed flagellum mutant showed that the filament structure acts as an adhesion. More work is needed to understand the involvement of the flagella regulon of R20291 in toxin expression. Our study shows that there are significant differences between different strains of *C. difficile* and that it is important to study phenomena in several strains before drawing general conclusions. As previously mentioned, differences in glycosylation might play an important role and go some way to explaining differences seen between aspects of motility, adherence and colonisation of different strains of *C. difficile*. It is also important to note that there are toxigenic, non-flagellated strains of *C. difficile*, for example PCR-ribotype 078, an epidemic strain with increasing importance. Interestingly these strains still possess the late flagellar genes, which might play a role in regulation.

## Materials and Methods

### Bacterial strains and growth conditions

All bacterial strains and plasmids used in this study are described in Table 1. *C. difficile* was routinely cultured on brain heart infusion (BHI) agar (Oxoid) or in BHI broth (Oxoid), with the following antibiotics where appropriate: thiamphenicol (15 µg ml^−1^), erythromycin (2.5 µg ml^−1^), lincomycin (20 µg ml^−1^), D-cycloserine (250 µg ml^−1^) and cefoxitin (8 µg ml^−1^) or cycloserine/cefoxitin antibiotic supplement (Fluka). Cultures were grown in an anaerobic cabinet (Don Whitney Scientific) at 37°C in an atmosphere of 10% CO_2_, 10% H_2_ and 80% N_2._ For cell toxicity assays and growth curves, Tryptose-yeast (TY) medium (3% [w/v] Bacto tryptose, 2% [w/v] yeast extract, and 0.1% [w/v] thioglycolate, adjusted to pH 7.4) was used. All *Escherichia coli* strains were routinely grown aerobically at 37°C on Luria-Bertani (LB) agar plates or in LB liquid culture in the presence of selective antibiotics where appropriate, with chloramphenicol (25 µg ml^−1^), kanamycin (50 µg ml^−1^) and ampicillin (100 µg ml^−1^).

**Table 1:** Strains and plasmids used in this study.

Name	Description	Source
**Bacterial strains**		
*E. coli* TOP 10	F– *mcr*A Δ(*mrr*-*hsd*RMS-*mcr*BC) Φ80*lac*ZΔM15 Δ*lac*X74 *rec*A1 *ara*D139 Δ (*ara leu*) 7697 *gal*U *gal*K *rps*L (StrR) *end*A1 *nup*G	Invitrogen
*E. coli* CA434	Conjugation donor	[30]
*C. difficile* 630	Wild-type, PCR-ribotype 012	Anaerobe Reference Laboratory, Cardiff, Wales
*C. difficile* 630Δ*erm*	Wild-type, PCR-ribotype 012, Erythromycin sensitive strain of *C. difficile* 630	[31]
*C. difficile* R20291	Wild-type, BI/NAP1/027 Stoke Mandeville (2004–2005) isolate	Anaerobe Reference Laboratory, Cardiff, Wales
CRG3357	*C. difficile* 630Δ*erm fliC*-515a::intron *ermB*	This study
CRG3356	*C. difficile* 630Δ*erm fliD*-560a:: intron *ermB*	This study
CRG3355	*C. difficile* 630Δ*erm flgE*-310s:: intron *ermB*	This study
CRG3354	*C. difficile* 630Δ*erm motA*-275a:: intron *ermB*	This study
CRG1509	*C. difficile* 630Δ*erm motB*-348s:: intron *ermB*	This study
CRG3353	*C. difficile* 630Δ*erm fliG*-663s:: intron *ermB*	This study
CRG3351	*C. difficile* R20291 *fliC*-430s:: intron *ermB*	This study
CRG3350	*C. difficile* R20291 *fliD*-121s:: intron *ermB*	This study
CRG3349	*C. difficile* R20291 *flgE*-311s:: intron *ermB*	This study
CRG1516	*C. difficile* toxin A^−^B^−^ double-mutant	[6]
CRG1987	*C. difficile* R20291-Δ*motB* (D23A)	This study
CRG3358	CRG3357 containing pMTL-SB1 (*fliC* complementation plasmid) in *C. difficile* 630*Δerm*	This study
CRG3360	CRG3356 containing *pMTL-SB2* (*fliD* complementation plasmid) in *C. difficile* 630*Δerm*	This study
CRG3362	CRG3355 containing *pMTL-SB3* (*flgE* complementation plasmid) in *C. difficile* 630*Δerm*	This study
CRG3359	CRG3351 containing *pMTL-SB1* (*fliC* complementation plasmid) in *C. difficile* R20291	This study
CRG3361	CRG3350 containing *pMTL-SB2 (fliD* complementation plasmid) in *C. difficile* R20291	This study
CRG2705-SB3	CRG311 containing *pMTL-SB3 (flgE* complementation plasmid) in *C. difficile* R20291	This study
CRG23-SB4	CRG23 containing *pMTL-SB4 (motB* complementation plasmid) in *C. difficile* R20291	This study
CRG23-SB5	CRG23 containing *pMTL-SB5 (motB* complementation plasmid) in *C. difficile* R20291	This study
**Plasmids**		
pMTL84151	Shuttle vector	[32]
pMTL84152	Shuttle vector	[32]
pMTL007C-E2	ClosTron plasmid	[25]
pMTL007C-E2:*fliC*-515a	ClosTron plasmid containing retargeted region to *fliC* at IS 515 (antisense oriented) for *C. difficile 630Δerm*	This study
pMTL007C-E2:*fliD*-560a	ClosTron plasmid containing retargeted region to *fliD* at IS 560 (antisense oriented) for *C. difficile 630Δerm*	This study
pMTL007C-E2:*flgE*-310s	ClosTron plasmid containing retargeted region to *flgE* at IS 310 (sense oriented) for *C. difficile 630Δerm*	This study
pMTL007C-E2:*motA*-275a	ClosTron plasmid containing retargeted region to *motA* at IS 275 (antisense oriented) for *C. difficile 630Δerm*	This study
pMTL007C-E2:*motB*-348s	ClosTron plasmid containing retargeted region to *motB* at IS 348 (sense oriented) for *C. difficile 630Δerm*	This study
pMTL007C-E2:*fliG*-663s	ClosTron plasmid containing retargeted region to *fliG* at IS 996 (sense oriented) for *C. difficile 630Δerm*	This study
pMTL007C-E2:*fliC*-430s	ClosTron plasmid containing retargeted region to *fliC* at IS 430 (sense oriented) for *C. difficile* R20291	This study
pMTL007C-E2:*fliD*-121s	ClosTron plasmid containing retargeted region to *fliD* at IS 121 (sense oriented) for *C. difficile* R20291	This study
pMTL007C-E2:*flgE*-310s	ClosTron plasmid containing retargeted region to *flgE* at IS 310 (sense oriented) for *C. difficile* R20291	This study
pMTL-SB1	pMTL84151 containing 873 bp *fliC* coding region and 100 bp upstream *fliC* promotor region	This study
pMTL-SB2	pMTL84151 containing 1,524 bp *fliD* coding region and 100 bp upstream *fliC* promotor region	This study
pMTL-SB3	pMTL84151 containing 984 bp *flgE* coding region and 100 bp upstream *fliC* promotor region	This study
pMTL-SB4	pMTL84151 containing 696 bp *motB* coding region and 100 bp upstream *fliC* promotor region	This study

### General molecular biology techniques

DNA manipulations were carried out according to standard techniques [33]. For DNA cloning and PCR analysis, *C. difficile* genomic DNA was prepared using quick Chelex resin-based or phenol-chloroform extraction techniques. DNA was purified from agarose gels using the QIAquick gel extraction kit (Qiagen, UK). Plasmids were isolated from *E. coli* using the plasmid mini-prep kit (Qiagen, UK) according to the manufacturer's instructions. PCR amplification was performed using Failsafe high fidelity DNA polymerase (Merck, UK) or Taq polymerase (Sigma) in accordance with the manufacturers' protocols.

### Construction of flagellar-associated mutants in *C. difficile* 630Δ*erm* and R20291

Flagellar target genes were insertionally inactivated in *C. difficile* 630Δ*erm* and R20291 using the ClosTron gene knock-out system as described previously [23], [25]. The mutants were verified by PCR screening and subsequent DNA sequencing using the primers shown in Table S1.

### Complementation of the flagellar mutants

For complementation of the *fliC* mutant, a 973-bp fragment encompassing the *fliC* open reading frame (873 bp) with the 100-bp 5′ noncoding region which likely encompasses the *fliC* promotor was amplified as a full-length DNA fragment with primers *Not*I-p*fliC*-630-F and *Xho*I-*fliC*-630-R and cloned using *Not*I and *Xho*I into the expression vector pMTL84151 to generate pMTL-SB1. The complementation primers were designed to allow cleavage of the *fliC* promoter and *fliC* gene as a full-length fragment. As the proteins encoded by *fliC*, *fliD*, *flgE* and *motB* are the same in 630Δ*erm* and R20291, only one plasmid (pMTL-SB1, pMTL-SB2, pMTL-SB3 and pMTL-SB4) was constructed for use in both mutants, respectively. To complement the *fliD* mutant, the *fliC* promoter was joined to the *fliD* gene by using splicing by overlap extension (SOEing) PCR [34] with the following primers: *Not*I-p*fliC*-R20291-F/*Nde*I-p*fliC*-*fliD*-R-SOE and *Nde*I-p*fliC*-*fliD*-F-SOE/*Xho*I-*fliD*-R to create two PCR products that were joined in a second step using the primers: *Not*I-p*fliC*-R20291-F/*Xho*I-*fliD*-R. The final product was cloned into the modular expression vector pMTL84151 resulting in pMTL-SB2. Likewise, SOEing PCR was used to construct a complementation plasmid with *flgE* (pMTL-SB3). For complementation of the *motB* (D23A) mutant, the 696-bp *motB* gene was amplified by standard PCR and cloned directly into the modular vector pMTL84152 (pMTL-SB4). All complementation plasmids were transferred into *C. difficile* via conjugation and transconjugants were verified by PCR.

### Southern blot

Mutants were verified by Southern blot using an intron specific probe. Genomic DNAs (2 µg) were digested with *Hin*dIII (NEB) overnight. The blots were carried out using a DIG high prime labelling and detection kit (Roche) according to the manufacturer's instructions.

### Motility assays

Swarming and swimming motility behaviours of *C. difficile* were studied by performing swarming and swimming motility assays as described [35] with minor modifications. Briefly, cultures of *C. difficile* were grown to mid-exponential phase for 8 h in BHI broth under anaerobic condition at 37°C. Motility plates were prepared by adding 25 ml of agar media into each plate and let to set for 2 h for swarm agar plates and overnight for swim agar plates. Soft agar plates were then air-dried for 15 min and transferred into the anaerobic workstation and allowed to pre-reduce for 4 h prior to inoculation with *C. difficile* strains. The swimming agar was made of BHI broth medium (37 g L^−1^) containing 0.3% (w/v) Difco bacto-agar and the swarming agar with 0.4% agar. To assay swimming plates were stab-inoculated with 3 µl of a mid-exponential growth culture of each strain at the same cell density and then incubated at 37°C for 48 h. Swimming motility was quantitatively determined by measuring the radius. To assay swarming plates were spot-inoculated with 3 µl of a mid-exponential growth culture of each strain and incubated as described above. Swarming motility was quantitatively determined by measuring the radius of the swarming zone. Motility assays were performed in six replicates for each strain and repeated independently three times.

### Transmission Electron Microscopy (TEM)

Electron microscopy was performed to examine the presence of flagellar structures on the surface of *C. difficile*. Cells were negatively stained using 0.5% Uranyl acetate (pH 4.5, Agar Scientific) on carbon formvar copper 200-mesh grids (Agar Scientific Ltd., UK). Briefly, cells from a mid-exponential *C. difficile* culture were adsorbed onto Formvar-coated copper grid for 5 min and the excess was carefully removed with blotting paper. This preparation was then fixed on the grid with 1% glutaraldehyde for 1 min. The grid was washed three times quickly with sterile distilled water for 10 s and cells were negatively stained with Uranyl Acetate for 30 s. The air-dried grid was visualized using the JOEL JEM1010 transmission electron microscope, operating at 80 kV.

### Cell culture

The human intestinal epithelial cell line Caco-2 and Vero cells (derived from African Green monkey kidney) were obtained from the American Type Culture Collection (ATCC, via Health Protection Agency, UK). Caco-2 and Vero cells were maintained at 37°C in 5% CO_2_ atmosphere in Dulbecco's modified Eagle's medium (DMEM) containing 10% (v/v) heat-inactivated foetal bovine serum (FBS), 1% (v/v) nonessential amino acids (NEAA) and 1% (v/v) antibiotic-antimycotic solution (containing penicillin [1 U ml^−1^], streptomycin [1 g ml^−1^], and amphotericin B as fungizone [2.5 mg ml^−1^]). For cell adherence assays, 12-well tissue-culture plates were seeded with 1×10^6^ Caco-2 cells per well and cells were grown for 9 days to obtain differentiated confluent monolayers. For cell cytotoxicity assay, 96-well tissue-culture plates was seeded at a density of approximately 0.25×10^5^ Vero cells per well and cells were grown for 48 h to obtain a confluent monolayer.

### Caco-2 cell adherence assay

The adherence of the *C. difficile* strains to differentiated human colonic enterocyte-like Caco-2 cells was assessed in the *in vitro* cell-culture adherence model under anaerobic condition using the protocol described recently by Barketi-Klai *et al*. [36] with the following modifications: 16 h cultures of *C. difficile* were harvested by centrifugation at 4000× g for 3 min. Cell pellets were resuspended gently in 1 m of the cell-line culture medium. Caco-2 cells were infected with 1×10^8^ cells ml^−1^ *C. difficile* by adding 1 ml of culture to each well of the 12-well tissue-culture plate and plates were then incubated for 1.5 h at 37°C under anaerobic condition. Initial bacterial counts were determined by plating onto selective BHI plates prior to infection. After incubation, monolayers were washed four times with sterile PBS to remove non-adherent bacteria. Then 1 ml of saponin 1% was added to each well, incubated for 10 min and attached bacteria were detached from cell monolayer by repeated pipetting. Bacterial counts at post-infection were determined by plating serial dilutions on selective BHI agar plates in triplicates. Colonies were counted after 24 h incubation. In parallel, uninfected monolayers (negative control) were collected by trypsinization and counted by trypan blue staining in order to express the adherence results as number of viable adherent CFU per one Caco-2 cell. Each adherence assay was performed in triplicate, and repeated at least three times in its entirety.

### Vero cell cytotoxicity assay

The kinetic of toxin secretion was examined in culture supernatants of *C. difficile* strains at different time points during growth (determined from early exponential to late stationary phase of growth) using Vero cell cytotoxicity assay as described previously by Kuehne *et al.* [6].

### Production of *C. difficile* culture supernatant filtrates

For the Vero cell cytotoxicity and GDH enzymatic activity determination assays, *C. difficile* strains were grown as follows. Overnight cultures in TY were used to set up a new starter culture using 1/100 inoculum. 2 ml of each *C. difficile* culture was collected at different time points during bacterial growth after recording OD_600_. Cells were harvested by centrifugation at 15000×g for 10 min at 4°C and the supernatant was filter-sterilized through 0.2 µm syringe filters (Sartorius, Germany). Filtered supernatant samples were then immediately frozen at −20°C to be used later for these assays.

### Preparation of *C. difficile* crude-cell extracts

For the glutamate dehydrogenase (GDH) enzymatic assay, whole cell protein extracts (soluble cytoplasmic fraction) were prepared at different time points during bacterial growth as follows: cell pellets from 2 ml *C. difficile* culture were harvested by centrifugation at 13000×g for 10 min, and lysed by resuspending in 200 µl Bugbuster 10× protein extraction buffer (Novagen UK) containing 2 µl of Benzonase Nuclease reagent (25 U µl^−1^, containing 50 mM tris-HCl, 20 mM NaCl, and 2 mM MgCl_2_, pH 8.0; Novagen UK) and 10 µl of Lysozyme solution (100 µg ml^−1^; Novagen UK). After 1 h incubation at room temperature with agitation, debris was pelleted by centrifugation at 13000×g for 20 min at 4°C and the crude cell extracts transferred to a new microfuge tube and stored at −20°C for later use.

### Glutamate dehydrogenase enzyme assay

GDH assay was performed using a previously described methodology [37] with minor modifications. Briefly, the GDH enzyme activity in *C. difficile* culture supernatant and soluble fraction was measured at 340 nm at 25°C with reaction mixture containing 0.333 ml of 300 mM Potassium Phosphate Buffer (pH 8.0), 0.167 ml of 300 mM L-glutamic acid (pH 7.5), 0.15 ml of 1 mM β-NAD, 0.333 ml of deionized water, and 0.017 ml of test sample. GDH enzyme (Sigma Chemical Co., UK) was used as the positive control. The reagents were added in the following order: Potassium Phosphate Buffer; L-glutamic acid; β-NAD; deionized water and finally the reaction was started by adding 6 U ml^−1^ of GDH [EC 1.4.1.3] as positive control or tested sample. The GDH activity was measured spectrophotometrically by recording the increase in absorbance at 340 nm for 5 min and the total absorbance was obtained by using the maximum linear rate for both tested samples and controls. GDH activity was calculated using the molar extinction coefficient for NAD(P) as 6.22 mM^−1^×cm^−1^. Enzyme specific activity was defined as one unit of GDH enzyme required to oxidate 1.0 mmole of L-glutamate to α-ketoglutarate per min using NAD(H) or NADP(H) as cofactors, at pH 8.0 and 25°C.

### RNA extraction and reverse transcription

These procedures were carried out essentially as described previously by Lyras *et al.* [5] with the following modifications. Total RNA was extracted from *C. difficile* grown to early stationary phase (16 h) in TY broth in four independent replicates for each strain. RNA stabilization was achieved by using RNAprotect Bacteria Reagent and an RNeasy Mini Kit (Qiagen) as described by the manufacturer's instructions. RNA isolation was then carried out using the FastRNA pro Blue extraction Kit (MP Biosciences), followed by phenol-chloroform-isoamyl alcohol (25∶24∶1) treatment and precipitated with ethanol according to the manufacturer's instructions. The total-RNA was dissolved in 50 µl of RNase-free water (DEPC-treated water) and DNase I digestion was then carried out to remove contaminating DNA in RNA samples by adding TURBO DNase buffer and TURBO DNase (Ambion) and incubated at 37°C for 1 h, according to the manufacturer's instructions. The RNA was purified using the RNeasy spin column purification kit (QIAGEN) according to the manufacturer's instructions. RNA samples were eluted from the spin column in two volumes of 20 µl of DEPC-treated water. The concentration of RNA samples was measured using the nanodrop spectrophotometer and stored at −80°C. The integrity and quality of extracted *C. difficile* RNA was determined using the Prokaryote Total RNA Pico assay by the Agilent 2100 bioanalyzer according to the manufacturer's instructions before use in RT-PCR. The absence of contaminating DNA in extracted RNA samples was confirmed by PCR with *fliC* gene specific primers. First-stranded cDNA synthesis was carried out by reverse transcription on 2 µg template RNA using Omniscript RT kit (Qiagen, UK) with random hexamer primers (Promega, UK) according to manufacturer's specifications. The cDNA samples were then purified using Qiaquick columns (Qiagen, UK).

### qRT-PCR (reverse transcriptase) analysis of *tcdA* gene expression

Real-time quantitative reverse transcriptase PCR (qRT-PCR) experiments were performed using the AB7500 cycler real-time PCR instrument. The qRT-PCR primers were designed and reactions were carried out using the SYBR Green Master Mix (Ap Biosciences, UK) with cDNA as template, as described by Lyras *et al.* [5]. PCR were performed on duplicate cultures and results were evaluated using *rpoA* as the endogenous control.

### Gnotobiotic mouse models

Animal care and animal experiments were carried out in strict accordance with the Committee for Research and ethical Issues of the International Association for the study of pain (IASP). The animal experimentation protocol was approved by the Animal Welfare Committee of the Paris Sud University and animal experiments were performed according to the University Paris Sud guidelines for the husbandry of laboratory animals. C3H/HeN germ-free (6–8 weeks old) and human microbiota-associated mice, obtained from INRA of Jouy-en-Josas (ANAXEM, France), were housed in sterile isolators provided with sterilized bedding and received standard nutrients sterilized by irradiation, and water sterilized by autoclaving. Prior to infection with *C. difficile* strains, sterility of axenic mice was confirmed by testing faecal culture of all tested animals. Faecal pellets were collected from mice, homogenised and serial dilutions were plated on BHI agar plates. Following 24 h incubation, plates showing no growth of bacteria indicated that tested animals were considered germ-free. Cultures of *C. difficile* for challenge were prepared for each mouse model according to the method described recently by Barketi-Klai *et al.* [36].

For the independent challenges (monoxenic mouse model), single infections of 1×10^8^ *C. difficile* were performed by oral gavage route on groups of 6 axenic mice. For the co-challenge (competition colonisation assay) (dixenic mouse model), mice were co-infected by oral gavage with 1×10^8^ bacterial CFU comprised of 1∶1 ratio of wild-type and mutant. Human Microbiota Associated mice (HMA) were dosed with 3 mg of amoxicillin/clavulanic acid (8/1: v/v) by oral gavage on 8 consecutive days to disrupt the intestinal microbiota. 24 h after the last antibiotic administration, each mouse was challenged with 1×10^8^ bacterial CFU. In these mouse models of colonisation, two parameters were studied as a surrogate for determining the level of *C. difficile* intestinal colonisation, these were:

1. **Intestinal implantation (** ***C. difficile*** **faecal shedding).** The concentration of *C. difficile* CFU recovered in faeces during the course of infection (kinetic of faecal shedding) was determined by homogenising and plating on *C. difficile* selective agar medium (supplemented with antibiotics, where appropriate).
2. **Caecal adherence.** The *C. difficile* caecal colonisation was studied by determining the level of *C. difficile* associated to the caecal wall of mice. Seven days after *C. difficile* challenge, mice were euthanized, dissected to excise the entire caecum of each mouse. Each caecum was rinsed in sterile PBS and weighed. The caecum of each mouse was harvested by homogenising using the ultra-Turrax apparatus (IKA-Labortechnik, Staufen, Germany) for 1 min at 12000*× g* using and diluted in PBS to obtain a final concentration of 10 mg ml^−1^. Ten-fold serial dilutions were then cultured on BHI agar plates (supplemented with antibiotics, where appropriate). Colonies were counted after 48 h incubation at 37°C under anaerobic condition.

### Statistical analysis

Statistical analysis was performed using StatEL and GraphPad Prism software. Data were analysed by Mann-Whitney test (mice experiments), Student's *t*-test, one-way analysis of variance ANOVA, followed by Dunnett's multiple comparison test (cytotoxicity and GDH assays). A statistically significant difference was considered to be p values of <0.05.

## Supporting Information

Figure S1 **Schematic representation of PCR screenings of putative ClosTron integrants in the** ***fliC*** **gene (a) and PCR screening (b).**
(TIF)

Figure S2 **Construction of the non-motile paralyzed flagellated** ***motB*** **(D23A) point mutant. Schematic representation (a), PCR screen (b), phenotypic characterization (c).**
(TIF)

Figure S3 **Adherence of the** ***C. difficile*** **630 flagella mutants to Caco-2 cells.**
(TIF)

Figure S4 **The role of** ***C. difficile*** **630Δ** ***erm*** **flagella in mouse models.**
(TIF)

Figure S5 **Cytotoxicity assay of** ***C. difficile*** **630 flagella mutants.**
(TIF)

Table S1 **Oligonucleotides used in this study.**
(DOCX)
