# Supplementary figures and images for: The Role of Flagella in Clostridium difficile Pathogenesis: Comparison between a Non-Epidemic and an Epidemic Strain
Source: PLoS One. 2013 Sep 23;8(9):e73026. doi: 10.1371/journal.pone.0073026 (PMC3781105; doi:10.1371/journal.pone.0073026)

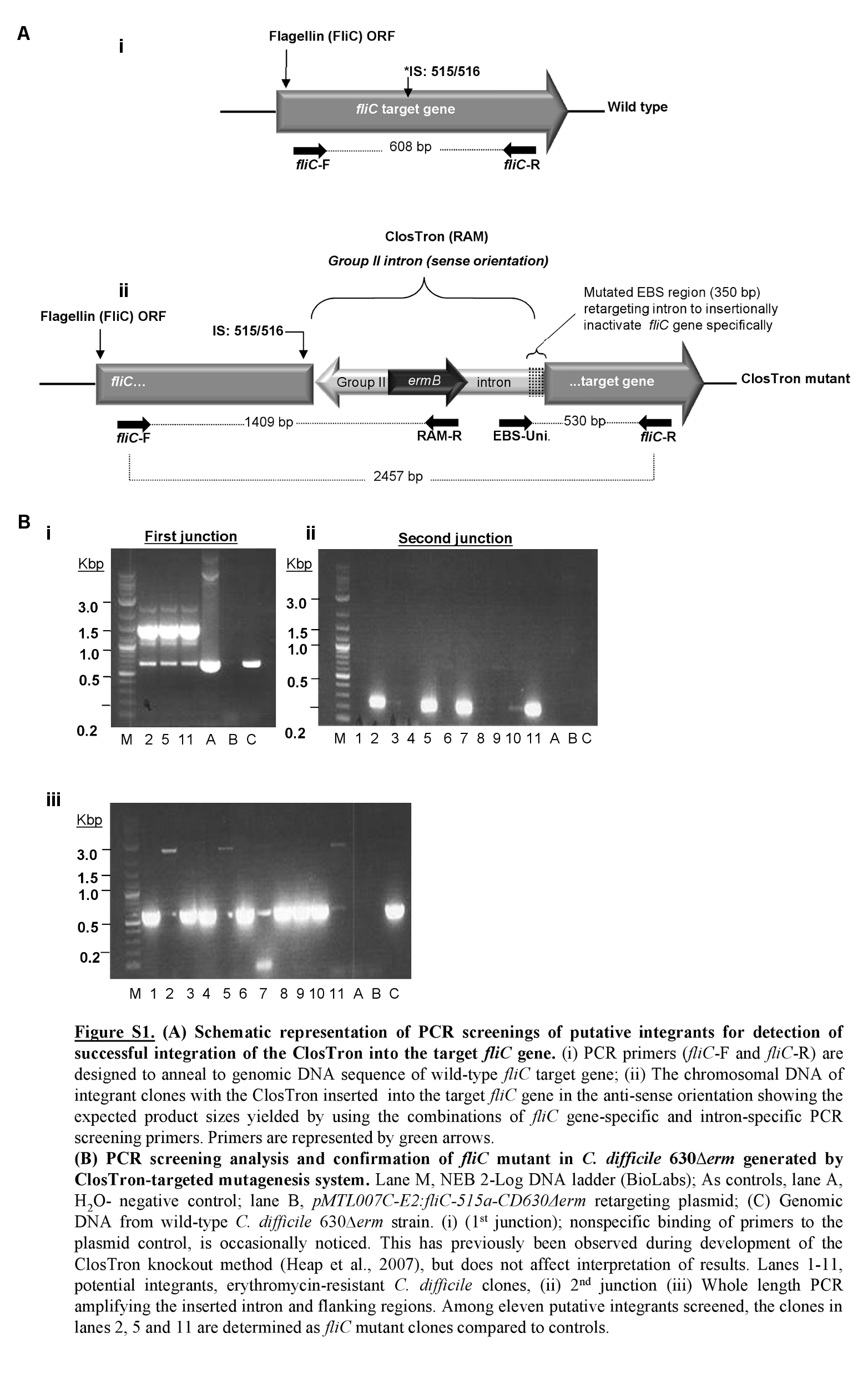

Supplement: Figure S1 — Schematic representation of PCR screenings of putative ClosTron integrants in the fliC gene (a) and PCR screening (b). (TIF) [file pone.0073026.s001.tif]

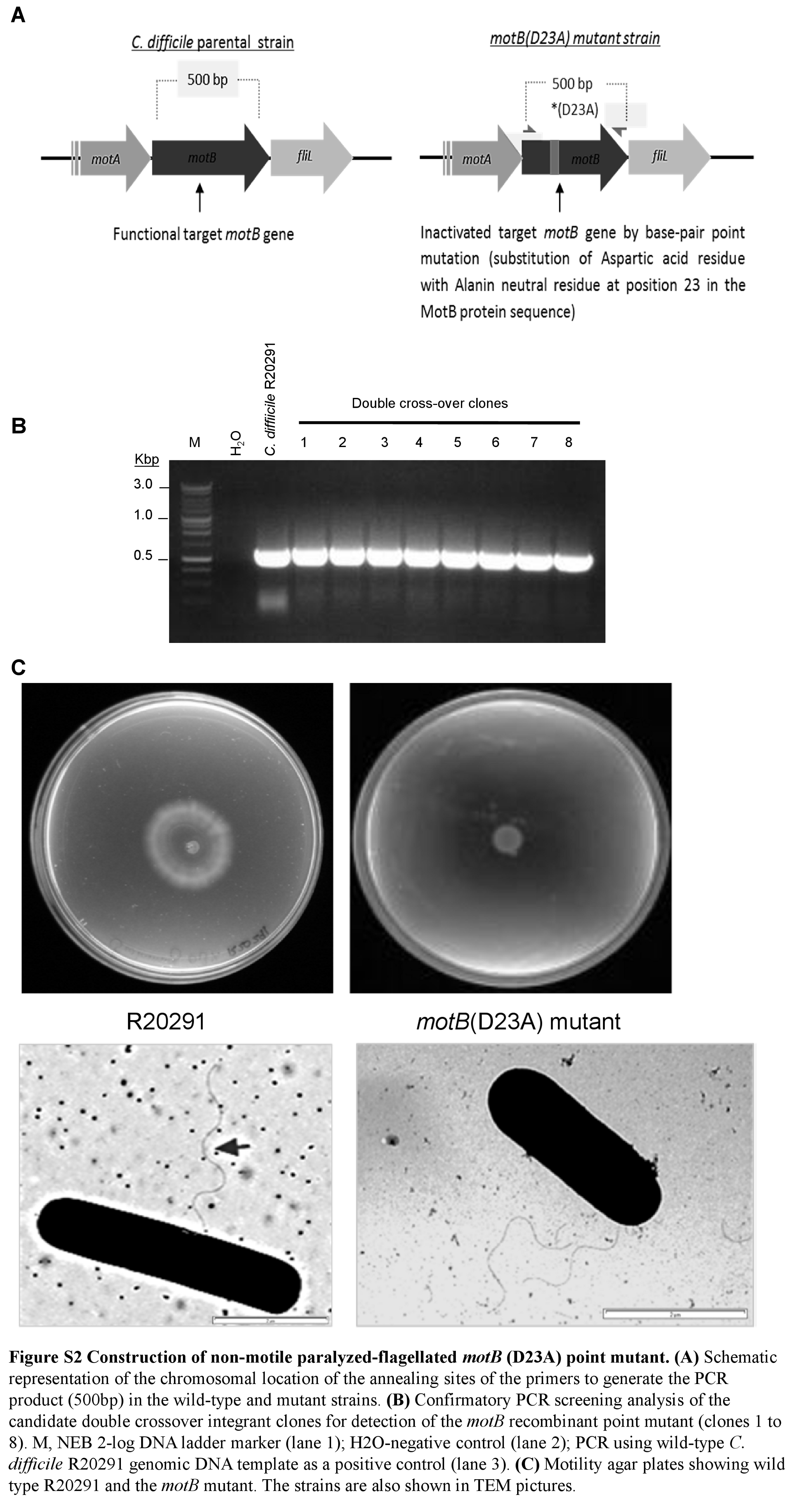

Supplement: Figure S2 — Construction of the non-motile paralyzed flagellated motB (D23A) point mutant. Schematic representation (a), PCR screen (b), phenotypic characterization (c). (TIF) [file pone.0073026.s002.tif]

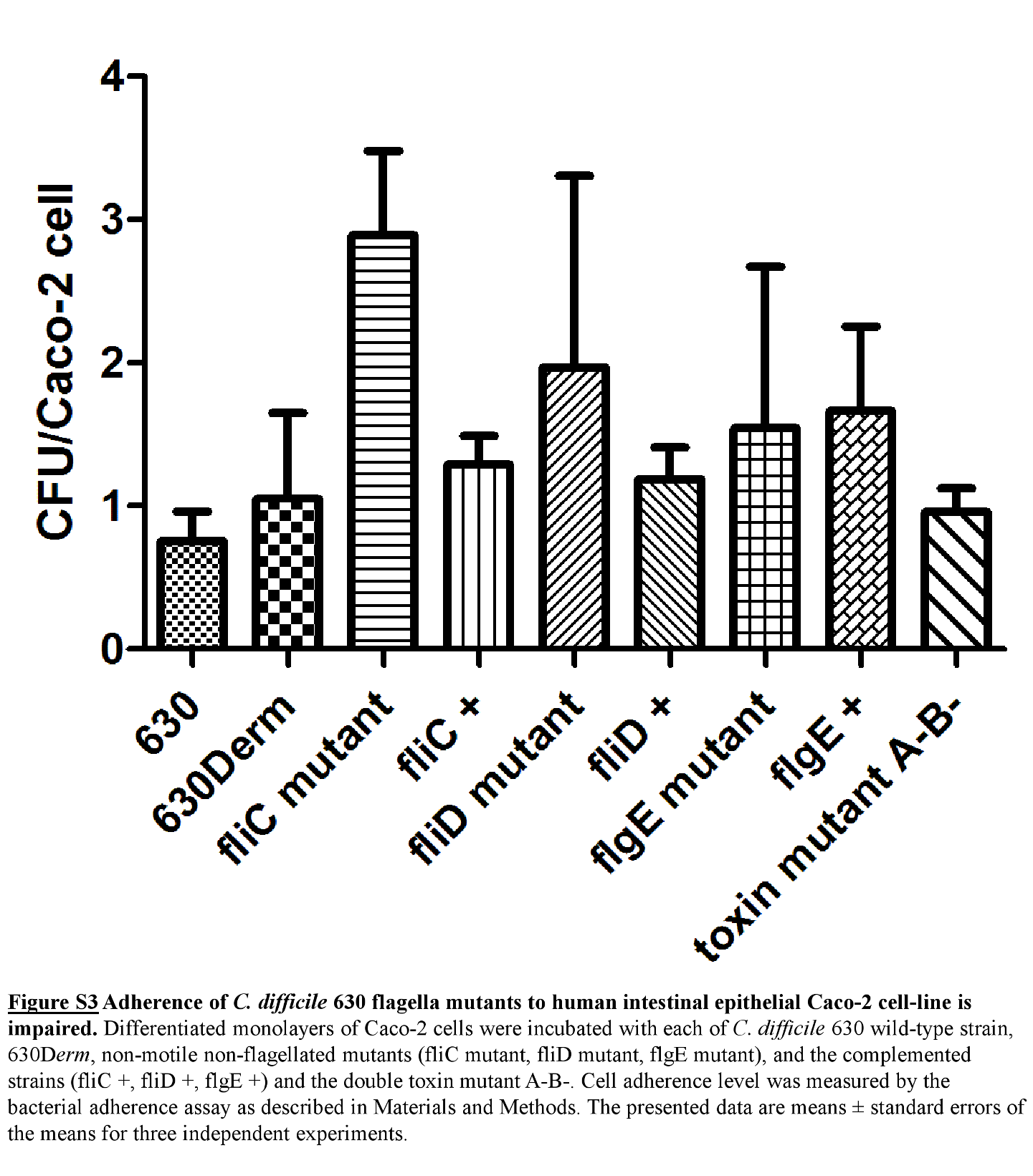

Supplement: Figure S3 — Adherence of the C. difficile 630 flagella mutants to Caco-2 cells. (TIF) [file pone.0073026.s003.tif]

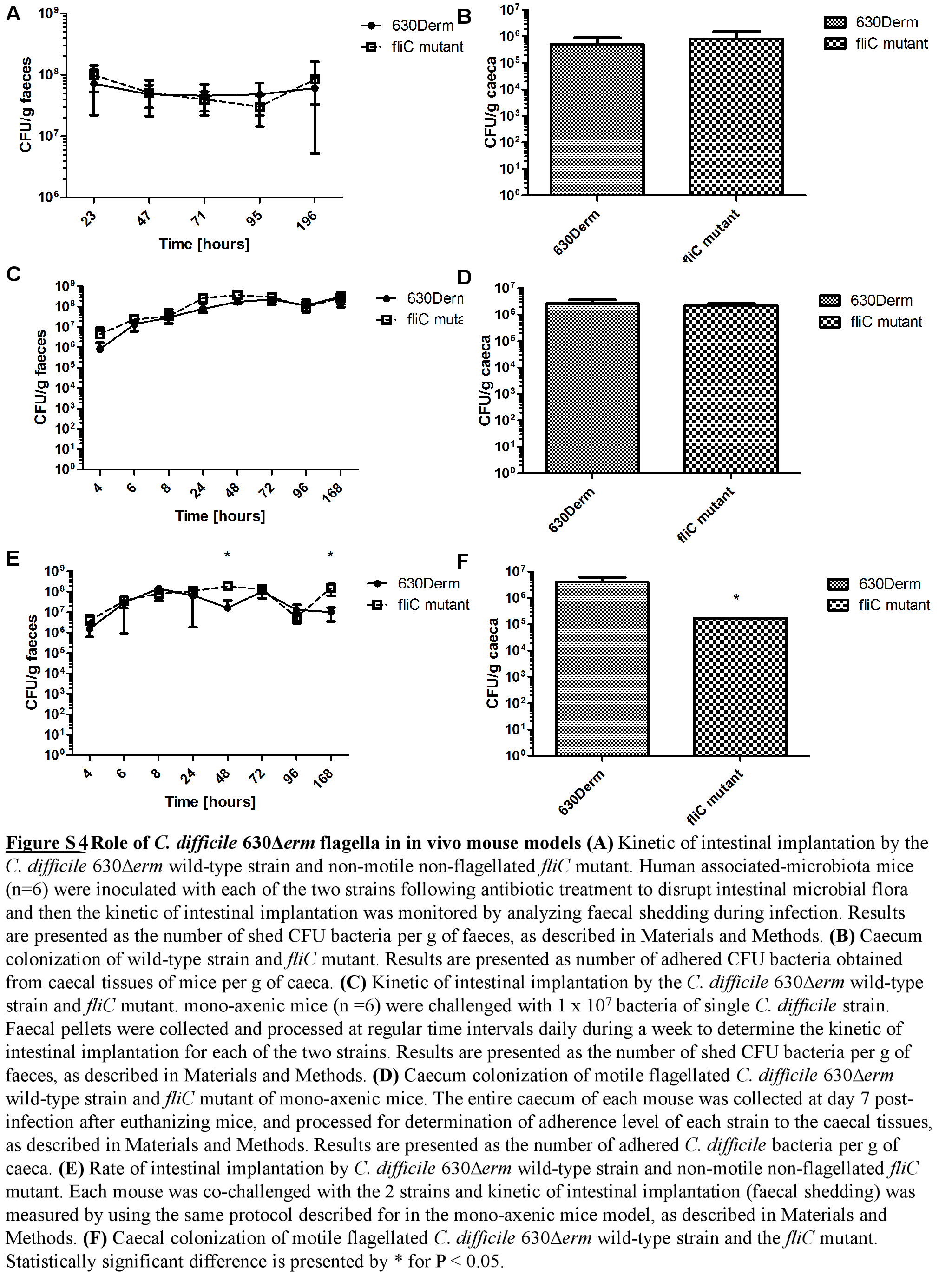

Supplement: Figure S4 — The role of C. difficile 630Δ erm flagella in mouse models. (TIF) [file pone.0073026.s004.tif]

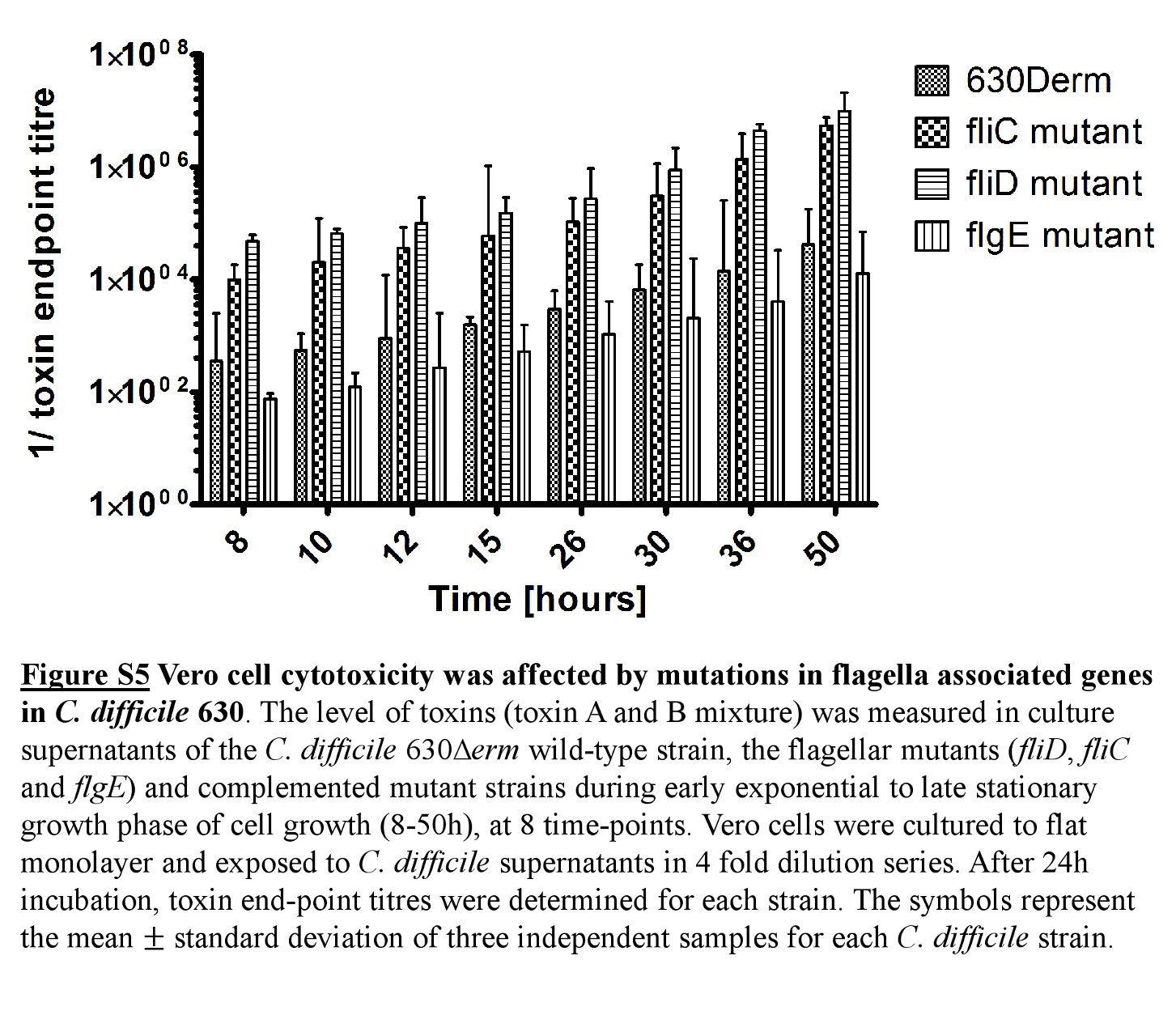

Supplement: Figure S5 — Cytotoxicity assay of C. difficile 630 flagella mutants. (TIF) [file pone.0073026.s005.tif]
