# Supplementary material for: The Role of Flagella in Clostridium difficile Pathogenesis: Comparison between a Non-Epidemic and an Epidemic Strain
Source: PLoS One. 2013 Sep 23;8(9):e73026. doi: 10.1371/journal.pone.0073026 (PMC3781105; doi:10.1371/journal.pone.0073026)
Supplement: Table S1 — Oligonucleotides used in this study. (DOCX) [file pone.0073026.s006.docx]

Supplementary data to

**The role of flagella in *Clostridium difficile* pathogenesis: Comparison of a non-epidemic and an epidemic strain.**

Soza T. Baban^≠1^, Sarah A. Kuehne^≠1^, Amira Barketi-Klai^2^, Kim R. Hardie^1^, Imad Kansau^2^, Anne Collignon^2^, Nigel P. Minton^1*^

^≠^ These authors contributed equally to this work.

**TABLE S1. Oligonucleotides used in this study**

| **Name** | **Sequence (5’ - 3’)** |
| --- | --- |
| **ClosTron primers:** |  |
| EBS universal | GTTTACTGAACGCAAGTTTCTAATTTCG |
| ErmRAM-F | CCCACTATTATTATTTTTATCAATATA |
| ErmRAM-R | GCGACTCATAGAATTATTTCCTCCCG |
| Cdi*-fliC-*515\|516a-IBS | AAAAAAGCTTATAATTATCCTTAATTATCCTTGCAGTGCGCCCAGATAGGGTG |
| Cdi*-fliC-*515\|516a -EBS1d | CAGATTGTACAAATGTGGTGATAACAGATAAGTCCTTGCAGTTAACTTACCTTTCTTTGT |
| Cdi*-fliC-*515\|516a -EBS2 | TGAACGCAAGTTTCTAATTTCGGTTATAATCCGATAGAGGAAAGTGTCT |
| Cdi*-fliD*-560\|561a-IBS | AAAAAAGCTTATAATTATCCTTAGTCTGCTTTCTTGTGCGCCCAGATAGGGTG |
| Cdi*-fliD*-560561a-EBS1d | CAGATTGTACAAATGTGGTGATAACAGATAAGTCTTTCTTGATAACTTACCTTTCTTTGT |
| Cdi*-fliD*-560\|561a-EBS2 | TGAACGCAAGTTTCTAATTTCGATTCAGACTCGATAGAGGAAAGTGTCT |
| Cdi*-flgE*-309\|310s-IBS | AAAAAAGCTTATAATTATCCTTAGGATACCTTCCAGTGCGCCCAGATAGGGTG |
| Cdi*-flgE*-309\|310s-EBS1d | CAGATTGTACAAATGTGGTGATAACAGATAAGTCCTTCCAGTTAACTTACCTTTCTTTG |
| Cdi*-flgE*-309\|310s-EBS2 | TGAACGCAAGTTTCTAATTTCGATTTATCCTCGATAGAGGAAAGTGTCT |
| Cdi*-motA*-275\|276a-IBS | AAAAAAGCTTATAATTATCCTTACCTTCCTTTCTAGTGCGCCCAGATAGGGTG |
| Cdi*-motA*-275\|276a-EBS1d | CAGATTGTACAAATGTGGTGATAACAGATAAGTCTTTCTAGCTAACTTACCTTTCTTTGT |
| Cdi*-motA*-275\|276a-EBS2 | TGAACGCAAGTTTCTAATTTCGGTTGAAGGTCGATAGAGGAAAGTGTCT |
| Cdi*-motB*-348\|349s-IBS | AAAAAAGCTTATAATTATCCTTAGGGATCTTACTAGTGCGCCCAGATAGGGTG |
| Cdi*-motB*-348\|349s-EBS1d | CAGATTGTACAAATGTGGTGATAACAGATAAGTCTTACTAGATAACTTACCTTTCTTTGT |
| Cdi*-motB*-348\|349s-EBS2 | TGAACGCAAGTTTCTAATTTCGATTATCCCTCGATAGAGGAAAGTGTCT |
| Cdi*-fliG*-663\|664s-IBS | AAAAAAGCTTATAATTATCCTTAGGAGTCTCTACCGTGCGCCCAGATAGGGTG |
| Cdi*-fliG*-663\|664s-EBS1d | CAGATTGTACAAATGTGGTGATAACAGATAAGTCTCTACCTTTAACTTACCTTTCTTTGT |
| Cdi*-fliG*-663\|664s-EBS2 | TGAACGCAAGTTTCTAATTTCGATTACTCCTCGATAGAGGAAAGTGTCT |
| Cdi*-fliC*-429\|430s-IBS | AAAAAAGCTTATAATTATCCTTATCTACCGAAATAGTGCGCCCAGATAGGGTG |
| Cdi*-fliC*-429\|430s-EBS1d | CAGATTGTACAAATGTGGTGATAACAGATAAGTCGAAATAAGTAACTTACCTTTCTTTGT |
| Cdi*-fliC*-429\|430s-EBS2 | TGAACGCAAGTTTCTAATTTCGGTTGTAGATCGATAGAGGAAAGTGTCT |
| Cdi*-fliD*-120\|121s-IBS | AAAAAAGCTTATAATTATCCTTAAAACACGAACAAGTGCGCCCAGATAGGGTG |
| Cdi*-fliD*-120\|121s-EBS1d | CAGATTGTACAAATGTGGTGATAACAGATAAGTCGAACAACATAACTTACCTTTCTTTGT |
| Cdi*-fliD*-120\|121s-EBS2 | TGAACGCAAGTTTCTAATTTCGGTTTGTTTCCGATAGAGGAAAGTGTCT |
| Cdi*-flgE*-309\|310s-IBS | AAAAAAGCTTATAATTATCCTTAGGATACCTTCCAGTGCGCCCAGATAGGGTG |
| Cdi*-flgE*-309\|310s-EBS1d | CAGATTGTACAAATGTGGTGATAACAGATAAGTCCTTCCAGTTAACTTACCTTTCTTTG |
| Cdi*-flgE*-309\|310s-EBS2 | TGAACGCAAGTTTCTAATTTCGATTTATCCTCGATAGAGGAAAGTGTCT |
| Cdi*-motB*-348\|349s-IBS | AAAAAAGCTTATAATTATCCTTAGGGATCTTACTAGTGCGCCCAGATAGGGTG |
| Cdi*-motB*-348\|349s-EBS1d | CAGATTGTACAAATGTGGTGATAACAGATAAGTCTTACTAGATAACTTACCTTTCTTTGT |
| Cdi*-motB*-348\|349s-EBS2 | TGAACGCAAGTTTCTAATTTCGATTATCCCTCGATAGAGGAAAGTGTCT |
| *fliC*-515a-630-F | GCAGAAGGTTCGTTAGAAGAAACTGG |
| *fliC*-515a-630-R2 | GCTTGAGCAAGCATTGATTGTGATGC |
| *fliD*-121s-630-F | CCAGTAAGAGTTACAGGCC |
| *fliD*-121s-630-R | CCAGCTGAGCCTTTTGCTAC |
| *fliD*-560a-630-F | CAACAGTTTCCTCCAAATGCTAGTGG |
| *fliD*-560a-630-R | CCTTGGTCATCAGTTACATCAGCTTC |
| *flgE* 310s-630-F | gtactatatgcaagtgctccaacagc |
| *flgE* 310s-630-R- degenerate | gctttatattccccatcataaatcaYacc |
| *motA*-275a-630-F | GCCGCAAAAGGAAGTAGTTCAGGG |
| *motA*-275a-630-R | CCCAAAAGCAGGTGCAAAGGTTCC |
| *motB*-348s-630-F | GGACAAGTATATTTAGTACGGG |
| *motB*-348s-630-R | CCCAATTGGATTCATGTGTACTTG |
| *fliG*-663s-CD630-F | GCACATATGGAACCAGAAAATGGGGC |
| *fliG*-663s-CD630-R | GCATCTGAAATCGCATCATCCACACC |
| *fliC*-430s-R20291-F | CCAAGCAGGAAGAAACGTTCAAGATGG |
| *fliC*-430s-R20291-R | GCTGCATCTGTTCCAGAAGTTCC |
| *fliD*-121s-R20291-F | GAGTTACAGGTCTTTCAGG |
| *fliD*-121s-R20291-R | GGAACTGTCGAACAATAGG |
| *motB*-348s-R20291-F | GGACAAGTATATTTAGTACGGG |
| *motB*-348s-R20291-R | CCCAATTGGATTCATGTGTACTTG |
| Spofdx-F1 | GATGTAGATAGGATAATAGAATCCATAGAAAATATAGG |
| pMTL007-R1 | AGGGTATCCCCAAGTTAGTGTTAAGTCTTGG |
| **Mutant complementation** | |
| M13-F | actggccgtcgttttaca |
| M13-R | caggaaacagctatg |
| *Not*I-p*fliC*-630-F | ACTGCGGCCGCAGTTATAGATTAACTTGTCCG |
| *Xho*I-*fliC*-630-R | GAATAAAAAAGAAAGGATAAGGCTTTGCCTCGAGGACAG |
| *Not*I-p*fliC*-R20291-F | CTGGCAGCGGCCGCGCTTACTAAACAAGTGAACAC |
| *Nde*I-p*fliC*-*fliD*-F-SOE | GGAGGGTAAAATACATATGTCAAGTATAAGTCCAG |
| *Nde*I-p*fliC*-*fliD*-R-SOE | CTATACTAAGGAGGGTAAAATACATATGTCAAGTATAAGTCC |
| *Xho*I-*fliD*-R | TATCTCGAGCATAATTAATTACCTTGTGCTTGTG |
| *Nde*I-*fliD*-F | ATACATATGTCAAGTATAAGTCCAGTAAGAG |
| *Nde*I-pfliC-*flgE-*R-SOE | GCTTTTATCATATGTATTTTACCCTCCTTAGTATAG |
| *Nde*I-pfliC-flgE-F-SOE | GGAGGGTAAAATACATATGATAAAAGCAATGTATTCAGG |
| *Nde*I-*flgE-*F | ATACATATGATAAAAGCAATGTATTCAGGTG |
| *Xho*I-*flgE*-R | TATCTCGAGCCTACTATCTCTTAAGATTTATTATTTC |
| *Nde*I-*motB*-F | ATACATATGGCGCGTAAAAAAAATAAGAAAAAGG |
| *Xho*I-*motB*-R | ATACTCGAGCCTCTTCCTCCTAGTCAATAG |
| *Nde*I-p*fliC*-*motB*-R-SOE | CGCGCCATATGTATTTTACCCTCCTTAGTATAG |
| *Nde*I-p*fliC*-*motB*-F-SOE | GGAGGGTAAAATACATATGGCGCGTAAAAAAAATAAG |
| ***C. difficile* 630Δ*erm* specific** | |
| 11870 | GGATAGAAATACTCGTCAACAG |
| 12870 | ATTTTTTATTTTTAGGAGTCATA |
